# Frequency of Intradialytic Complications in Patients of End-Stage Renal Disease on Maintenance Hemodialysis

**DOI:** 10.7759/cureus.12641

**Published:** 2021-01-11

**Authors:** Muhammad Ali, Ayesha Ejaz, Hina Iram, Shafique A Solangi, Abdul Manan Junejo, Sagheer Ahmed Solangi

**Affiliations:** 1 Department of Nephrology, Fazaia Ruth Pfau Medical College, Karachi, PAK; 2 Department of Nephrology, Jinnah Postdraduate Medical Centre, Karachi, PAK; 3 Department of Nephrology, Jinnah Postgraduate Medical Centre, Karachi, PAK; 4 Department of Internal Medicine, Jinnah Postgraduate Medical Centre, Karachi, PAK

**Keywords:** hemodialysis, arteriovenous fistula, hypotension, seizures, dialyzer reaction

## Abstract

Introduction

Hemodialysis is a vital management option for end-stage renal disease (ESRD) patients. With adequate hemodialysis, patients can have a good quality of life but complications do occur during the session, which can be minor to life-threatening. The objective of this study was to assess the frequency of acute complications during this procedure.

Material and methods

An observational, cross-sectional study was conducted at Jinnah Postgraduate Medical Centre, Karachi, Pakistan. Patient data were collected about access, comorbid conditions, frequency and duration of hemodialysis, and intradialytic complications. Those with acute kidney injury were excluded.

Results

There was a total of 94 patients, with a mean age of 45.51±13.29 years, of which 62 (66%) were males and 32 (34%) were females. Diabetes mellitus was the most common cause of ESRD (47.9%, n=45). Patients on twice and thrice-weekly sessions were 51 (54.3%) and 43 (45.7%), respectively. The most common complication was hypotension (28.7%), followed by hypertension (17%), and nausea/vomiting (11.7%). The arteriovenous fistula was the most common access used (75.5%, n=71). Most patients were found to be on hemodialysis for more than five years (51.1%, n=48).

Conclusion

Blood pressure changes are critical while performing hemodialysis, just like we found hypotension as the most common intradialytic complication in our results, followed by hypertension. Others were fever, muscle cramps, and nausea/vomiting. a prospective follow-up study shall be done to have comparative and long-term results related to the acute and chronic complications of dialysis.

## Introduction

Globally, the prevalence of kidney diseases has increased in the last two decades, currently standing at 11-13% [1]. This might be due to the increasing burden of diabetes mellitus (DM) (the leading cause of kidney disease), hypertension, glomerular diseases, renal stone diseases, and the use of over-the-counter medications. Treatment for end-stage renal disease (ESRD) is renal replacement therapy (RRT) in the form of dialysis (either hemodialysis or peritoneal) or renal transplantation. Hemodialysis is one of the most common medical facilities used in the world, with more than two million patients enrolled for regular hemodialysis annually [2]. The entire procedure takes three to four hours, given thrice a week in the developed world and twice a week in the developing countries [3].

They are likely to have comorbid disorders like ischemic heart disease, peripheral vascular disease, cerebrovascular disease, and chronic obstructive pulmonary disease, all of which are associated with increased morbidity and mortality. Though hemodialysis is a life-saving treatment for ESRD patients, it is one of the relatively safe procedures having a mortality of 1/75,000 treatments. However, it is associated with various complications, some of which are acute, occurring during or immediately after the session, while others are chronic complications [4].

Although the etiology of these complications is multifactorial and poorly understood, some are machine and procedure-related factors like the type of dialyzer, dialysis solution (acetate or bicarbonate), conductivity, blood flow, volume, and rate of ultrafiltration, an anticoagulant used, and contamination of Reverse Osmosis water. We call them technical complications. These are rare nowadays because of the advancement in technologies and equipment of hemodialysis. Other causes are patient-related factors like the primary disease, comorbid conditions, medications, intradialytic weight gain, frequency, length of the session, and duration of hemodialysis [5].

Among the acute complications reported worldwide, the most common is hypotension (25-55%). The National Kidney Foundation-Kidney Disease Outcomes Quality Initiative defines hypotension as a decrease in systolic blood pressure (SBP) >20 mm Hg or a decrease in mean arterial pressure by 10 mm Hg [5]. Other complications are arrhythmias (50%), nausea/vomiting during and after the session (15%), muscle cramps (20%), headache, chest pain (5%), back pain (5%), hypertension, fever, and chills due to dialyzer reaction [6,7]. There is a difference in the frequencies and pattern of acute complications during hemodialysis in different regions of the world, depending upon genetic and ethnic variations, and the primary cause of ESRD. Moreover, few local studies reported differences in the percentage of complications as well [8].

The prevalence of ESRD in Pakistan is 14.6% [9]. Moreover, with the highest growing incidence of chronic diseases ultimately leading to ESRD, it is crucial to evaluate the pros and cons of the management options [9]. The purpose of this study is to determine the various complications that most commonly occur during hemodialysis so that precautionary steps can be sorted out. This will enhance the knowledge about the complications of hemodialysis in the medical staff as well as the patients to help them in deciding for treatment.

## Materials and methods

This is a cross-sectional study conducted at the Department of Nephrology, Jinnah Postgraduate Medical Center, Karachi, Pakistan from March to August 2020, after approval from the ethical review board of the institution (N0.F.2-81-IRB/2020-GEN/49836/JPMC). Informed consent was taken from the patients for enrollment in the study.

All the patients above the age of 18 years on hemodialysis for more than three months were included. Pregnant patients, those with acute kidney injury, dementia or disorientation, or those on hemodialysis for less than three months were excluded. A total of 94 patients were registered in the specified time duration.

Patient data was filled on a self-designed questionnaire regarding age, gender, cause of ESRD, frequency, and duration of hemodialysis. Blood samples were taken for hemoglobin, serum calcium, phosphate, uric acid, albumin, iron, creatinine, urea, vitamin D, and intact parathyroid hormone (iPTH) as per schedule and recommendations. Adequacy of hemodialysis was calculated as urea reduction ratio (URR) and single pool (Sp) Kt/V (where Kt/V shows dialysis adequacy by incorporating dialyzer clearance of urea-K, dialysis time-t, and volume of distribution of urea-V). Each patient’s number and type of complications were noted.

Data were analyzed by IBM Statistical Package for the Social Sciences (SPSS) Statistics for Windows, version 21.0 (IBM Corp., Armonk, NY). Mean with standard deviations were calculated for age, blood sample values, URR, Sp Kt/V, and duration of hemodialysis, while frequency and percentages were calculated for all the categorical ones. Stratification was done according to gender, dialysis access, duration, and sessions per week. Post-stratification, the chi-square test was applied, with a p-value of <0.05 as statistically significant.

## Results

We enrolled 94 patients of ESRD that were on hemodialysis, with a mean age of 45.51±13.29 years, of which 62 (66%) were males and 32 (34%) were females.

Most were on three times per week hemodialysis schedule (45.7%). Over six months, a total of 5544 sessions were performed, where 3096 were in the thrice-weekly schedule and 2448 were in the twice-weekly schedule. The majority were found to be on hemodialysis for more than five years (51.1%, n=48). Native arteriovenous fistula (AVF) was the most common type of access used (75.5%) (Table 1).

**Table 1:** Patient hemodialysis characteristics

	Frequency	Percentage
Session-week		
Thrice per week	43	45.7%
Twice per week	51	54.3%
Access		
Arteriovenous fistula	71	75.5%
Arteriovenous graft	7	7.4%
Tunnel catheter	7	7.4%
Temporary central catheter	9	9.6%

DM was the most common cause of ESRD (47.9%), followed by hypertension (17.0%), chronic glomerulonephritis (10.6%), and bilateral small size kidneys (10.6%) (Table 2).

**Table 2:** Cause of end-stage renal disease

	Frequency	Percentage
Hypertension	16	17.0%
Pregnancy-related cause	3	3.2%
Bilateral small size kidneys	10	10.6%
Chronic glomerulonephritis	10	10.6%
Contrast-induced nephropathy	2	2.1%
Diabetes mellitus	45	47.9%
Renal stone	6	6.4%
Myeloma	2	2.1%

The mean values of blood sample investigations are given in Table 3.

**Table 3:** Blood laboratory values Sp Kt/V: single pool Kt/V Note: Kt/V shows dialysis adequacy by incorporating dialyzer clearance of urea (K), dialysis time (t), and volume of distribution of urea (V).

	Mean	Standard deviation
Vitamin D (ng/dL)	25.47	13.445
Intact parathyroid hormone (pg/dL)	576.59	584.284
Hemoglobin (mg/dL)	9.29	1.400
Uric acid (mg/dL)	8.16	15.413
Iron (mcg/dL)	93.63	44.456
Calcium (mg/dL)	8.11	0.824
Phosphate (mg/dL)	5.67	1.705
Albumin (mg/dL)	3.33	0.538
Creatinine (mg/dL)	7.95	2.507
Urea reduction ratio (%)	64.79	3.980
Sp Kt/V	1.14	0.124

**Figure 1:**
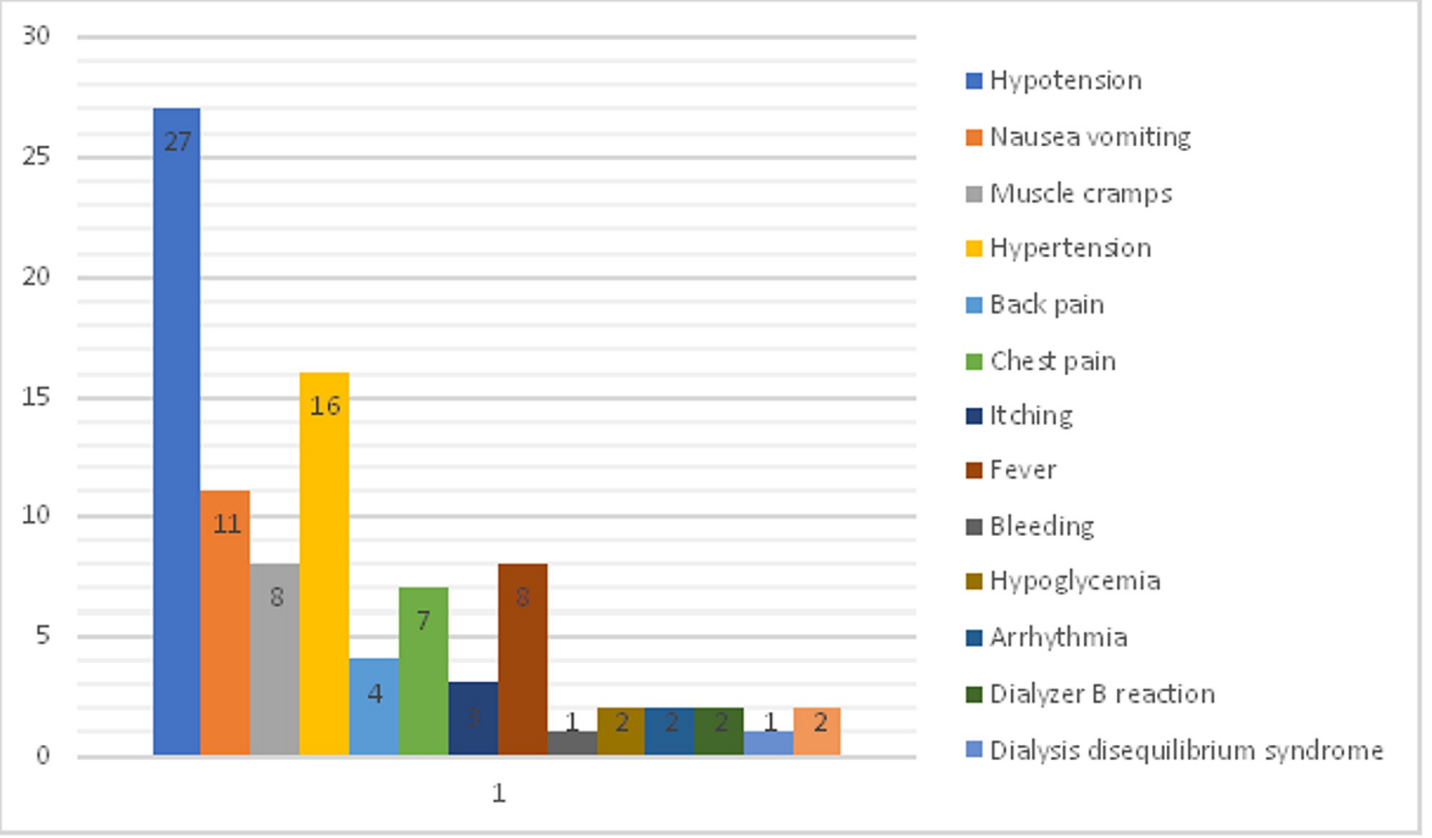
Graphic presentation of intradialytic complications

Hypotension was found to be the most common complication (28.7%, n=27), followed by hypertension (17.0%, n=16), nausea/vomiting (11.75%, n=11), fever (8.5%, n=8), and muscle cramps (8.5%, n= 8) (Figure 1).

Complications were then assessed according to gender, dialysis access, duration, and sessions per week as shown in Table 4.

**Table 4:** Complications according to gender, dialysis access, duration, and sessions per week (n=94)

	Complications														Total	P-Value
	Hypotension (n=27)	Nausea/Vomiting (n=11)	Muscle Cramps (n=8)	Hypertension (n=16)	Back pain (n=14)	Chest Pain(n=7)	Itching (n=3)	Fever (n=8)	Bleeding (n=1)	Hypoglycemia (n=2)	Arrhythmia (n=2)	Dialyzer Reaction B(n=2)	Dialysis Disequilibrium Syndrome (n=1)	Seizures (n=2)		
Gender																0.046
Male	13	8	6	14	3	3	3	7	1	1	0	2	1	0	62	
Female	14	3	2	2	1	4	0	1	0	1	2	0	0	2	32	
Access																0.103
Arteriovenous fistula	22	7	6	11	3	6	2	7	1	2	1	1	0	2	71	
Arteriovenous graft	1	0	0	4	0	0	1	0	0	0	0	0	1	0	7	
Tunnel catheter	2	3	0	0	1	0	0	0	0	0	1	0	0	0	7	
Temporary central catheter	2	1	2	1	0	1	0	1	0	0	0	1	0	0	9	
Duration since the start of dialysis																0.007
<6 months	0	0	0	1	0	0	0	1	0	0	0	0	0	0	2	
1 year	0	0	0	0	0	0	1	0	0	0	0	0	0	0	1	
2 year	1	0	1	0	0	0	0	0	0	0	0	0	1	0	3	
3 year	7	1	1	3	2	1	1	2	0	0	1	2	0	1	22	
4 year	4	2	0	6	1	1	1	2	0	1	0	0	0	0	18	
5 year	15	8	6	6	1	5	0	3	1	1	1	0	0	1	48	
Sessions per week																
Thrice per week	14	5	4	7	1	4	1	5	0	0	0	2	0	0	43	
Twice per week	13	6	4	9	3	3	2	3	1	2	2	0	1	2	51	

## Discussion

RRT in patients with ESRD is given either as renal transplantation or dialysis. Hemodialysis is the most common modality used worldwide. In-center hemodialysis is cost-effective, increasing socioeconomic burden as well as the psychological disorders. In the last few decades, there is advancement and modernization in hemodialysis machines, technologies, and substances used for the session to deliver the best quality of hemodialysis, like bicarbonate solution, online hemodiafiltration, and continuous RRT techniques [8]. Hypotension is the most common intradialytic complication (28.7%, n=27) reported in our study.

The exact mechanism for intradialytic hypotension is not fully understood and is believed to be multifactorial due to the excess intradialytic weight gain, rapid fluid removal, cardiovascular system impairment, infections, allergic reactions, high dialysate temperature, low sodium, and medications used before the session [10]. Islam et al. in 2017 reported hypotension as the most common complication in 12.62% of patients [11], while Mahmood et al. found it in 24% [12]. A meta-analysis from Netherland found the same in 12% of patients [13], while Habas et al. found it in 15.2% [14].

The second most common intradialytic complication reported in our study was hypertension (17.0%, n=16), defined as a rise in SBP >10 mm Hg above the pre-dialysis value during, just at the end, or shortly after the session [15]. Intradialytic hypertension occurs most likely due to the low ultra-filtration rate, excessive weight gain, sympathetic overactivity, and clearance of antihypertensive medicine during hemodialysis. A secondary analysis of a randomized controlled trial compared the effects of blood pressure changes on mortality and hospitalization rate and concluded that increased SBP during hemodialysis had higher chances of mortality and increased hospitalization compared with that of hypotension [16]. In a local study, Shaikh et al. also reported hypertension as the second most common cause (3.54%) [17]. Islam et al. reported hypertension in 8.25% of patients [11]. However, Mahmood et al. found intradialytic hypertension only in 1% of patients [12]. Inrig et al. and Buren et al. found it in 10% and 21.3%, respectively [16,18].

Nausea/vomiting was found in 11.75% of patients in this study, i.e., higher than what was reported by another local study (3.22%) [17]. Internationally a prevalence of 2-15% was seen [18].

Another significant complication reported in our data collection was fever (8.5%, n=8) that occurred mostly in patients with temporary catheters due to line infection, first use dialyzers, membrane incompatibility, and sepsis. Another local study also reported the same [11], while an Indian study found fever in 14.4% of patients during hemodialysis [4]. A much lower percentage was seen in the works of Mahmood et al. (1-4%) and Rashid et al. (1.5%) [12,17].

Chest pain was found in 7.4% of our study population, a much lower percentage compared to that by Islam et al. (1.47%) [11], Rashid et al. (0.9%) [17]. However, Mahmood et al. reported it in 15% [12].

Hemodialysis is a lifeline for ESRD, a comparatively safe procedure with modern machines and technology, Intradialytic complications as enumerated in the results of this study can be prevented by proper counseling, less intradialytic weight gain, strict measures of infection control, appropriate dialysate temperature, adjustment of antihypertensive medications, and strict vigilance of vital signs during hemodialysis. These results are limited by the fact that the sample size cannot be representative of the entire population. Secondly, a prospective study should be done to evaluate and compare the number and type of complications (both acute and chronic).

## Conclusions

Blood pressure changes are critical while performing hemodialysis, just like we found hypotension as the most common intradialytic complication in our results, followed by hypertension. Others were fever, muscle cramps, and nausea/vomiting. a prospective follow-up study shall be done to have comparative and long-term results related to the acute and chronic complications of dialysis.
